# Inferential Composition Control of a Distillation Column Using Active Disturbance Rejection Control with Soft Sensors

**DOI:** 10.3390/s23021019

**Published:** 2023-01-16

**Authors:** Fahad Al Kalbani, Jie Zhang

**Affiliations:** School of Engineering, Merz Court, Newcastle University, Newcastle Tyne NE1 7RU, UK

**Keywords:** distillation columns, inferential control, active disturbance rejection control, principal component regression

## Abstract

This paper presents the integration of active disturbance rejection control (ADRC) with soft sensors for enhancing the composition control performance in a distillation column. Static and dynamic soft sensors are developed to estimate the top and bottom product compositions using multiple tray temperatures. In order to cope with the collinearity issues in tray temperature measurements, static and dynamic principal component regression is used in developing the soft sensors. The soft sensor outputs are introduced as the feedback signals to ADRC. This control scheme is termed as “inferential ADRC control”. Static control offsets are eliminated through mean updating in the soft-sensor models. The effectiveness of the proposed control scheme is demonstrated on a benchmark simulated methanol-water distillation column.

## 1. Introduction

In the last decades, the proficient and efficient use of energy has become a highly significant issue in the industrial sector since the prices of energy as well as environmental awareness are continuously increasing. Thus, industry is highly interested in approaches for minimizing the energy consumption in industrial processes [1]. Distillation is still one of the most commonly used and one of the most versatile separation methods for separating liquid mixtures in petrochemical and chemical industries accounting for about 25–40% of the energy usage in the sector. Due to its relatively low energy efficiency, this unit process is often one of the biggest energy consumers in industrial processes. When considering energy efficiency in any type of distillation columns, it is really necessary to account for the form of energy being consumed and the quality of cooling and heating required. Trade-offs exist between environmental impact, cost, energy sources and equipment requirements. Distillation columns consume a huge amount of energy for providing heat required to convert liquid to vapor and then condense the vapor back to liquid via the condenser. Distillation uses more than 40% of the amount of energy utilized in the refining and bulk chemical process industry and more than 90–95% of energy consumed in liquid separation and purification, and accounts for more than 3% of the energy consumption in the world [2]. Moreover, the capital investment of these distillation systems is indicated to be at least eight billion US dollars which can contribute to greater than 50% of both capital and plant operating costs in a typical chemical plant which can have a significant impact on the overall plant profitability [3]. The minimum energy expected to be consumed in distillation columns depends on various operation variables such as temperature spans and operating pressures and the optimization of these variables leads to reducing the energy demand while meeting product quality and quantity requirement [4]. It is very likely that distillation will continue to be the choice of liquid mixture separation for the next decade as it is still labelled “as the technique of choice for many current purification and separation operations” but it needs to make radical modifications and changes to reduce energy consumption. With the rising environmental concerns and growing energy awareness there is a need to minimize the energy use in all industry sectors [4].

Advanced control of distillation columns is one way of improving their energy efficiency. The control of distillation column must drive the product compositions as close to their desired set-points as possible in the faces of unexpected disturbances in feed flow rate and feed composition. However, it is quite difficult to get reliable and accurate product compositions economically on-line and without time delay. The time delay in most composition analyzers is typically between 10–20 min. Such large time delays cause poor control performance and degraded process operation because the effects of disturbances remain undetected for substantial periods of time. The most common alternative technique to product composition control using composition analyzers is indirect composition control through tray temperature control. However, single tray temperatures are not very accurate indicators of product compositions especially under disturbances. Therefore, one approach to overcome this issue is to implement inferential control with multiple tray temperatures in conjunction with advanced control scheme, such as the active disturbance rejection control (ADRC) scheme, to improve the overall control performance. Inferential control using soft sensors is capable of alleviating the issue of large measurement delays by using secondary outputs (tray temperatures) to infer the state of primary outputs (product compositions).

This paper is organized as follows: Section 2 gives an overview of ADRC and inferential control. A binary methanol–water separation column is presented in Section 3. Section 4 presents an inferential ADRC control strategy for binary distillation columns. Both static and dynamic soft sensors implementation for product composition using principal component regression (PCR) is presented in Section 5. Section 6 presents the control performance of inferential ADRC. The last section presents some concluding remarks.

## 2. An Overview of Active Disturbance Rejection Control and Inferential Control

### 2.1. Active Disturbance Rejection Control

Many industrial plants in the real world are not just time varying and nonlinear but also highly uncertain. The design of control systems for such plants has been the focus of much of the current improvements and developments under the umbrella of adaptive, robust and nonlinear control. However, most of the proposed control methods are based on the assumption that a fairly accurate mathematical model of the plant is available and due to their dependence and complexity on advanced analytical methodologies and mathematical model, these methods have certain limitations in engineering applications. According to the well-known control theorist Roger Brockett that if there is no uncertainty in the system, then feedback control is largely unnecessary [5].

Recognizing the vulnerability and sensibility of the reliance on accurate mathematical models of many modern control algorithms, there has been a gradual avowal over the years that active disturbance estimation is a practical alternative to accurate plant models. Moreover, if a disturbance exists in the plant and is represented by the discrepancy between the industrial plant and its model, then this disturbance can be estimated in real time. Then, the plant-model mismatch can be successfully and efficiently compensated for, making the model-based design tolerant of a considerable number of uncertainties. The main focal point in the close control of such plants is how unknown dynamics and external disturbance can be predicted or estimated.

**Figure 1:**
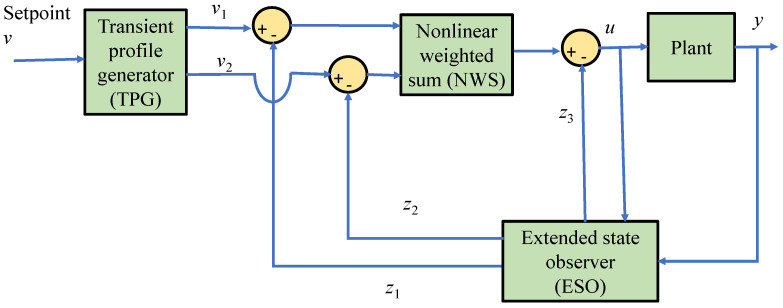
Structure of ADRC.

ADRC was introduced in 1995 by Prof. Jinqing Han at the Chinese Academy of Science [6,7,8,9]. However, most of the earlier papers are in Chinese and the concept of ADRC was first introduced into English literature in 2001 by Gao [10,11,12]. The methodology of ADRC has been in development for over two decades and has been utilized in various engineering applications. It has been considered as an alternative paradigm in control engineering to address and investigate non-linear and time variant systems [12]. ADRC is considered as an advanced form of principle of active control (PAC) and inherits its concept from the limitations of proportional–integral–derivative (PID) which are error computation, oversimplification of control law as the form of linear weighted sum (LWS), noise degradation associated with the derivative term and complications associated to the integral control term. The main advantages of ADRC are model independency and disturbance rejection [11,12]. Figure 1 shows the ADRC structure which consists of three main parts: transient profile generator (TPG), non-linear weighted sum (NWS), and extended state observer (ESO).

TPG proposed in [9] is a second order system that may produce smooth transition output process tracking the input set-point signal. Moreover, it is an effective technique to solve the conflict between avoiding overshoot and quickness in response of the controlled variable. Han [9] proposed that TPG could be constructed by using the following equation.

(1) $$\left\{\begin{array}{c} \dot{V_{1}}=V_{2}\\ \dot{V_{2}}=-rsign\left(V_{1}-V+\left.\frac{v_{2}\left|v_{2}\right|}{2r}\right)\right.\\ \dot{V_{2}}=fhan\left(v_{1}-v\left(t\right),v_{2},r,h_{0}\right) \end{array}\right.$$

In the above equation, V is the setpoint for the controlled variable, $V_{1}$ is the desired trajectory, $V_{2}$ is the derivative of the desired trajectory, *r* is sometimes called tracking speed, $h_{0}$ is the filtering factor, and fhan is the Han function [9]. It can be noticed that the value of the parameter *r* can be selected depending on the physical limitation of the plant. The speed of the transient profile can be slowed down or speeded up by selecting a suitable value of *r*.

Usually, the conventional PID control employs a linear combination of proportional (present), integral (accumulative) and derivative (predictive) of the tracking errors. Moreover, other possibilities of combinations that might be much more effective are ignored. In addition, it usually needs the strategy on trade-off between fastness and overshoot of the control response. In order to avoid this contradiction, Han [9] gives an alternative nonlinear function which depends on the magnitude of error signal to produce the control signal.

Systems are operating under different types of disturbances, among which the ones that have some impacts on the output signal are the most significant. As a result, the disturbances can be separated from the output signal by creating or defining new state which can be done by ESO. ESO generates the estimates of the unknown disturbances and unmeasured system states and then compensates them. Furthermore, ESO can enhance the system performance adaptability.

Consider the following 2nd order system [9]:

(2) $$\left\{\begin{array}{c} {\dot{x}}_{1}=x_{2}\\ {\dot{x}}_{2}=f\left(x_{1},x_{2},d_{e},t\right)+bu\\ y=x_{1} \end{array}\right.$$

where *y* is the system output, *u* is the manipulated variable for controlling *y*, and *f*(*x*_1_, *x*_2_, *d_e_*, *t*) is a multivariable function of the states *x*_1_ and *x*_2_, the undesired external disturbance *d_e_*, and time *t*. This function reflects the effect of the total disturbance *d_t_*(*t*). Using the total disturbance *d_t_*(*t*) as an additional state variable, Equation (2) can be organized as follows:

(3) $$\left\{\begin{array}{c} \begin{array}{c} {\dot{x}}_{1}=x_{2}\\ {\dot{x}}_{2}=x_{3}+bu \end{array}\\ \begin{array}{c} {\dot{x}}_{3}=d_{t}(t)\\ y=x_{1} \end{array} \end{array}\right.$$

The states *x*_1_(*t*) to *x*_3_(*t*) can then be estimated by an ESO and the estimated states are denoted as *z*_1_(*t*) to *z*_3_(*t*) respectively. By inspecting Figure 1 and in order to remove the impact of the total undesired disturbance on the controlled variable, the control law of the ADRC scheme can be written as:

(4) $$u=\frac{g-z_{3}(t)}{b_{0}}$$

where *g* is the desired closed loop dynamics, *z*_3_(*t*) is the estimate of the total disturbance *d_t_*(*t*), and *b*_0_ is an approximation of the parameter *b* in Equation (2).

### 2.2. Overview of Inferential Control

The increasing availability of a wide range of sensors and data acquisition systems has led to a corresponding rise in the amount of data that can be logged through the computer control and monitoring systems of industrial processes. Hardware sensors give information on the process operation in terms of process variables, such as pressures, temperatures and flow rates, and product quality variables, such as composition and polymer molecular weight. Such sensors for product quality variables can be utilized to provide information on the quality of the final product in order to certify that it satisfies the customer requirements. However, many product quality variables cannot be easily and economically measured. Such sensors like composition analyzers usually possess large measurement delay and they are usually expensive. In many cases, the main product quality indicators are generally obtained by off-line sample analysis in a scientific laboratory. On-line quality analyzers such as gas chromatography and Near-InfraRed (NIR) are typically expensive and usually incur high maintenance cost [13,14]. Furthermore, significant delays and discontinuity associated with slowly processed quality measurements and laboratory analysis of on-line analyzers may reduce the efficiency and effectiveness of control policies. Instead of product composition control using composition analyzer and NIR, tray temperature control is broadly used to indirectly control product compositions. Moreover, tray temperature measurements are economic, reliable and virtually without any measurement time delays. However, utilizing single tray temperature to characterize the product composition has some drawbacks such as column pressure variation and feed rate or composition variation can significantly affect the correlation between tray temperatures and product compositions. In industrial processing plants, such restriction and limitations can have a severe impact on product quality.

In an effort to overcome the problems encountered in product composition measurement, soft sensing or inferential estimation techniques have acquired momentum recently as viable alternatives to hardware sensors in on-line process monitoring and control [15]. In the last two decades, there has been rising interest and research in the development of soft sensors to provide regular on-line predictions of quality variables based on easy-to-measure process variables. Such soft sensors provide real time estimates of product quality variables and help to improve closed loop control performance and develop tight control policies [16]. A soft sensor can be considered as a mathematical model that generates reliable real time estimates of unmeasured variables from easy-to-measure process variables [16].

There are various advantages of soft sensors in the monitoring and control of industrial processes:

- They provide more insight into the process through catching the information hidden in data;
- They provide enhanced monitoring and control of industrial processes with the consequences of reducing environmental impact, enhancing productivity and energy efficiency, and improving business profitability through decreasing the production cost related to off-specification products;
- They can be simply implemented on existing hardware. Moreover, on-line model identification algorithms can be utilized to adapt the model when plant characteristics change; and
- They entail little or no capital costs such as installation cost, commissioning and management of the required infrastructure.

The design of soft sensors can be either by utilizing grey or black box identification approaches or on the basis of an analytical model. In the development of data-driven empirical model, least squares regression has been widely used. Nevertheless, when numerous input variables are used, this technique can become ineffective due to the strongly correlated nature of process variables. For instance, distillation column tray temperatures are closely correlated to each other and change together in the same pattern. Using linear regression techniques on such highly correlated process data leads to numerical errors due to close to singularity in the data covariance matrix. The common approach for tackling correlation problems is to select a few appropriate variables which are less correlated from each other [17,18,19]. However, this simple technique is not optimal because the information in the discarded measurements might enhance the model performance.

Brosilow and co-workers [17,18] introduced a composition estimator called the Brosilow Estimator in which flow rates and temperatures were used for predicting unmeasured disturbance and then the estimated disturbances were utilized to predict product compositions. However, in recent years, product composition estimators have been designed using partial least squares regression (PLS) [20,21]. Mejdell and Skogested [22] compared three linear model-based composition estimators of a binary distillation column. They briefed that good control performance might be reached with the steady state PCR (principal component regression) estimator. They found that the performance of the steady state PCR estimator is nearly good as the dynamic Kalman filter. Zhang [23,24] developed an inferential feedback control strategy for binary distillation composition control using PCR and PLS models. In these works, both top and bottom compositions are estimated via multiple tray temperature measurements and the estimated top and bottom product compositions are then used as feedback control signals.

## 3. A Binary Distillation Column for Methanol-Water Separation

**Figure 2:**
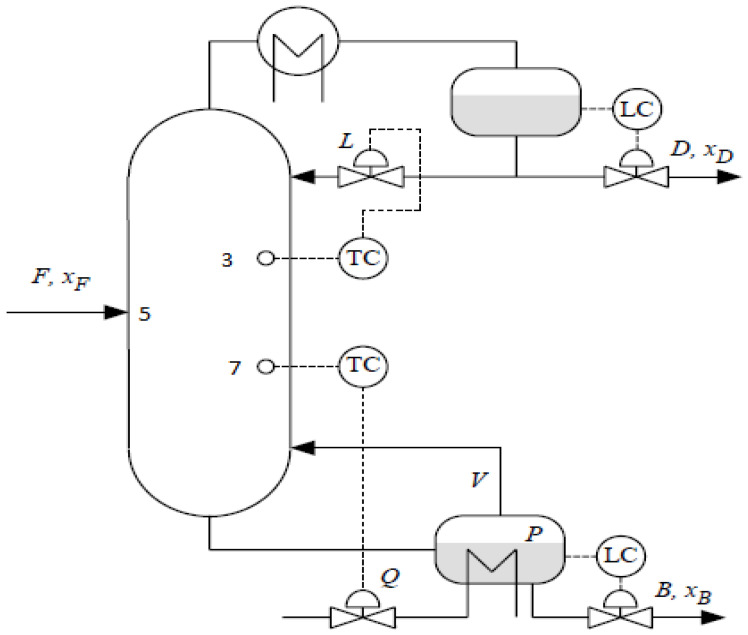
A binary distillation column with tray temperature control scheme.

The distillation column considered in this paper is a comprehensive nonlinear simulation of a methanol–water separation column which is based on the Wood and Berry’s column at University of Alberta in Canada. The schematic diagram of this distillation column is shown in Figure 2. The column has 10 trays including the re-boiler and the condenser. The feed stream enters the distillation column at the 5th tray. The following assumptions are used in the development of a rigorous mechanistic model: constant liquid holdup, negligible vapor holdup, and perfect mixing in each stage. The nominal operation data for this column are given in Table 1. The nominal set-points of the product compositions in this study are the distillate at 93% and the bottom composition at 7%. A dynamic simulation program is developed in MATLAB based on the mechanistic model. In our previous study [25], we compared product composition control in this distillation column using ADRC and PID control. It is shown that ADRC gives better performance than PID for both setpoint tracking and disturbance rejection. However, the practical difficulty in product composition measurements is not considered in [25] and it is assumed that product composition measurements are available without time delays.

## 4. Inferential ADRC Scheme

**Figure 3:**
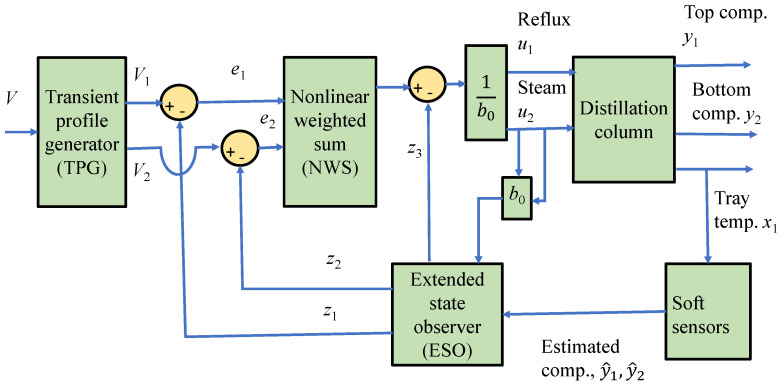
Inferential ADRC control scheme.

The proposed inferential ADRC scheme for distillation column product composition control is shown in Figure 3. It can be seen from Figure 3 that the top composition (*y*_1_) and bottom composition (*y*_2_) are taken as the primary controlled variables where the secondary measurements are tray temperatures (*x*). Moreover, the distillation column is subjected to two different disturbances which are feed flow rate and feed composition disturbances. In this control scheme, both top and bottom compositions are estimated via multiple tray temperature measurements through soft sensors and the estimated product compositions are then used as feedback signals for the composition controllers. The soft sensors are developed using PCR. It should be noted that for highly nonlinear processes, such as batch distillation processes, nonlinear soft sensors should be utilized [26,27,28,29]. In our earlier work [30], static PCR models are used. In the current work, the soft sensors are extended to using dynamic PCR models.

## 5. PCR Model-Based Software Sensors

**Figure 4:**
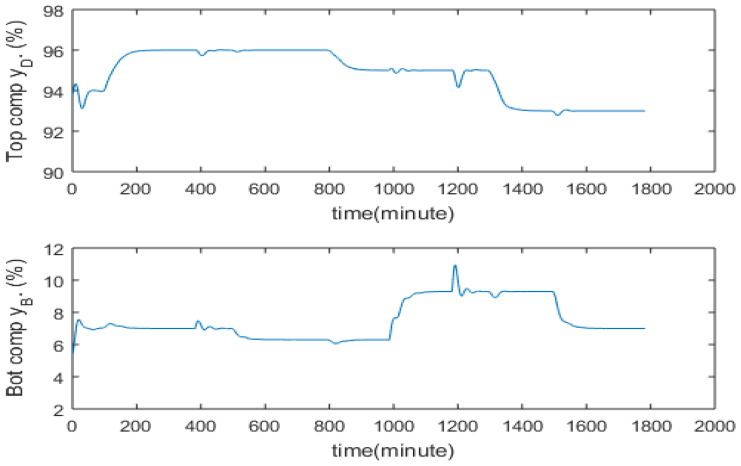
Top and Bottom product compositions.

**Figure 5:**
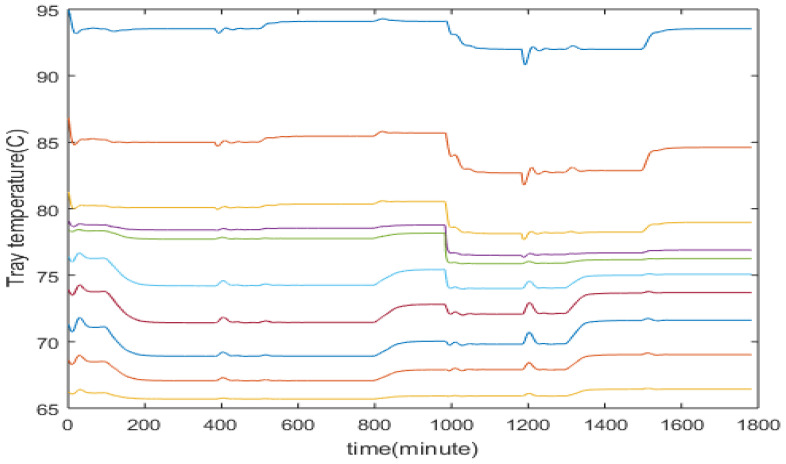
Tray temperatures.

In order to develop soft sensors, historical process operational data containing measurements of tray temperatures and product compositions are required. In this study, simulated process operation data are generated covering different operating conditions (with setpoint changes and disturbances). The data set for tray temperature and product composition in a real distillation column can be obtained from historical plant operation. Temperature measurement devices such as thermocouples are cheap and all tray temperatures can be easily monitored. Composition analyzers can be expensive and it might not be economically viable to install dedicated composition analyzers for each distillation column in a plant. In this case, the plant can put on temporary composition analyzers, e.g., hired ones, to a distillation column for the period of modelling campaign for the purpose of data collection. As shown in Table 1, the nominal operating point considered in this paper is that the top composition at 93% and the bottom composition at 7%. Simulated process operational data around this nominal operating are generated. Figure 4 shows the top and bottom product compositions while the corresponding secondary measurements of tray temperatures are shown in Figure 5. It can be seen from Figure 5 that a strong correlation exists among tray temperature measurements.

### 5.1. Static PCR Models

In the static PCR model based soft sensors, the product compositions at time *t* are estimated using the tray temperatures at time *t*. The soft sensors can be presented in the following form:

(5) $$y\left(t\right)=\theta_{1}T_{1}\left(t\right)+\theta_{2}T_{2}\left(t\right)+\cdots +\theta_{10}T_{10}\left(t\right)$$

where *y* denotes the estimated product compositions, *T*_1_ to *T*_10_ represent the tray temperatures from tray 1 to tray 10 respectively, *θ*_1_ to *θ*_10_ are corresponding model parameters, and *t* represents the discrete time. Before developing the soft sensors, the data are first scaled to zero mean and unit variance. The complete set of tray temperature and product composition data is divided into two sets: the training data set (samples 1 to 1189) and the testing data set (samples 1190 to 1982). PCR models with various numbers of principal components are developed on the training data and then tested on the testing data. The PCR model with the lowest error on the testing data is considered as having the suitable number of principal components and is taken as the final soft sensor.

Table 2 presents the sum of squared errors (SSE) of different PCR models on the training and testing data. It can be seen that the PCR model with six principal components gives the best performance for the top composition on the testing data and 10 principal components offers the best performance for the bottom compositions on the testing data. Therefore, six principal components are used in the top composition model and 10 principal components are used in the bottom composition model. The developed PCR models for top and bottom product compositions are as follows [30]:

(6) $$\begin{array}{c} y_{D}\left(t\right)=93+0.0450∆T_{1}\left(t\right)-0.0357∆T_{2}\left(t\right)-0.1304∆T_{3}\left(t\right)+0.1891∆T_{4}\left(t\right)-0.0345∆T_{5}\left(t\right)+0.0881∆T_{6}\left(t\right)\\ -0.3115∆T_{7}\left(t\right)-0.3255∆T_{8}\left(t\right)-0.0666∆T_{9}\left(t\right)-0.6737∆T_{10}\left(t\right) \end{array}$$

(7) $$\begin{array}{c} y_{B}\left(t\right)=7-0.3944∆T_{1}\left(t\right)+0.0718∆T_{2}\left(t\right)-0.2206∆T_{3}\left(t\right)+1.3567∆T_{4}\left(t\right)+0.2175∆T_{5}\left(t\right)+0.8840∆T_{6}\left(t\right)\\ -0.9850∆T_{7}\left(t\right)-0.8758∆T_{8}\left(t\right)-1.7598∆T_{9}\left(t\right)-0.7149∆T_{10}\left(t\right) \end{array}$$

where *y_D_* and *y_B_* represent the top and bottom compositions (wt%) respectively, and Δ*T* is the deviation of a tray temperature from its nominal mean value.

**Figure 6:**
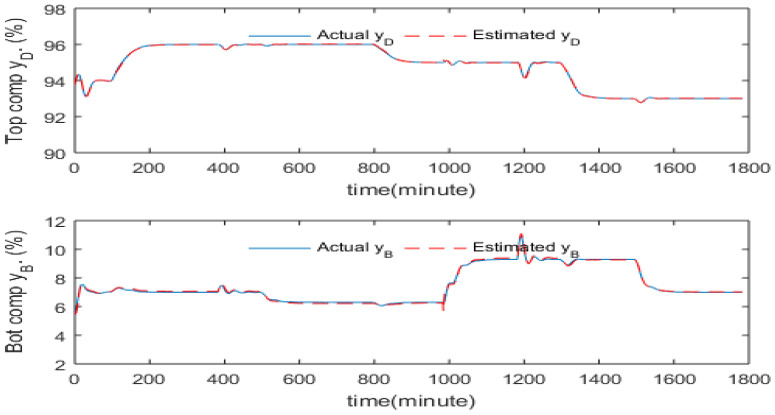
Predictions from the PCR model.

Figure 6 gives the PCR model predictions. It can be seen from this figure that the model predictions are very accurate, especially for the top product composition.

### 5.2. Dynamic PCR Models

The inferential estimation accuracy might be further enhanced and improved if dynamic PCR models are developed. In this paper, dynamic PCR models with orders ranging from one to seven were developed. The first order dynamic PCR models can be represented in the form below:

(8) $$y\left(t\right)=\theta_{1,1}T_{1}\left(t\right)+\theta_{1,2}T_{1}\left(t-1\right)+\theta_{2,1}T_{2}\left(t\right)+\theta_{2,2}T_{2}\left(t-1\right)+\cdots +\theta_{10,1}T_{10}\left(t\right)+\theta_{10,2}T_{10}\left(t-1\right)$$

Data partition and data scaling are the same as in developing static PCR models presented earlier. The suitable numbers of principal components were once again specified by the least SSE on the testing data. Table 3 presents the number of principal components and the corresponding SSE values on the testing data of these dynamic PCR models.

**Figure 7:**
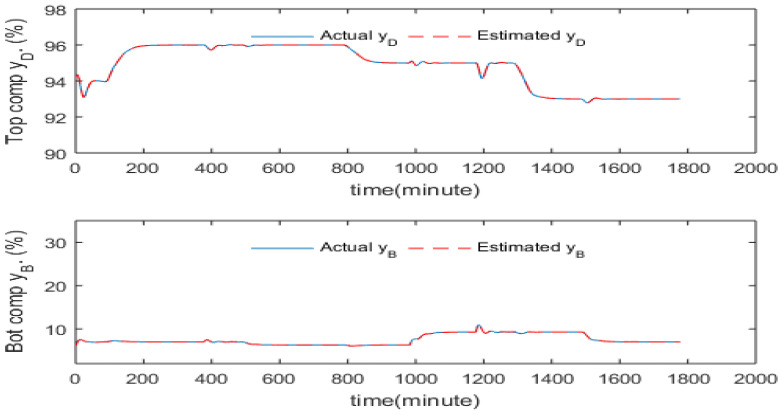
Model predictions from the 5th order dynamic PCR model.

It can be seen that the dynamic PCR models significantly enhance the estimation accuracy compared to the static PCR model especially at third order, fourth order, fifth order, and sixth order models. All these four models have been compared, discussed and investigated in terms of SSE values. The differences in SSE values between these four models are not significant. Hence, the fifth order dynamic PCR model is used and integrated with the ADRC scheme to estimate the top and bottom product compositions. The estimations from the 5th order dynamic PCR model are shown in Figure 7. Again, in this figure the solid lines represent the actual measured compositions while the dashed lines represent the corresponding model estimations.

It can be seen that the dynamic PCR models significantly improve the estimation accuracy compared to the static PCR model especially at the 3rd to 6th order models. All these four models have been compared, discussed and investigated in terms of SSE values. The differences in SSE values between these four models are not significant. Hence, the 5th order dynamic PCR model is used and integrated with the ADRC scheme to control the top and bottom compositions. The estimations from the 5th order dynamic PCR model are shown in Figure 7. Again, in this figure the solid lines represent the actual measured compositions while the dashed lines represent the corresponding model estimations.

**Figure 8:**
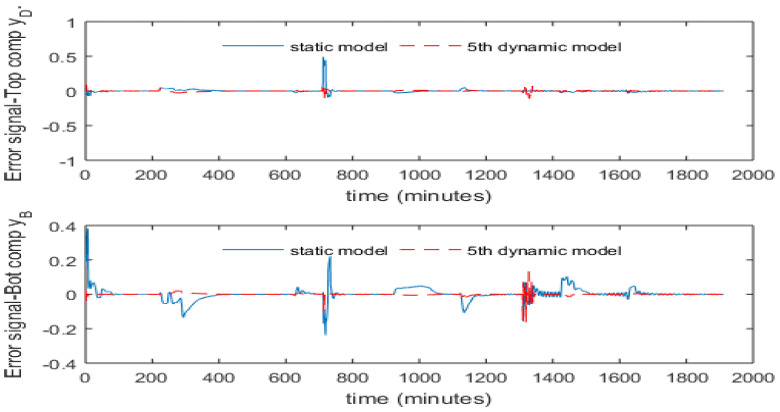
Model prediction errors.

The model parameters of the 5th order dynamic PCR models are given in Appendix A, where Table A1 gives the model parameters for the top composition and Table A2 gives the model parameters for the bottom composition. Figure 8 presents the estimation errors for both the static and the 5th order dynamic PCR model. It can be seen that the 5th order dynamic PCR model gives better prediction performance than the static model.

## 6. Inferential ADRC Scheme Based on PCR Models

**Figure 9:**
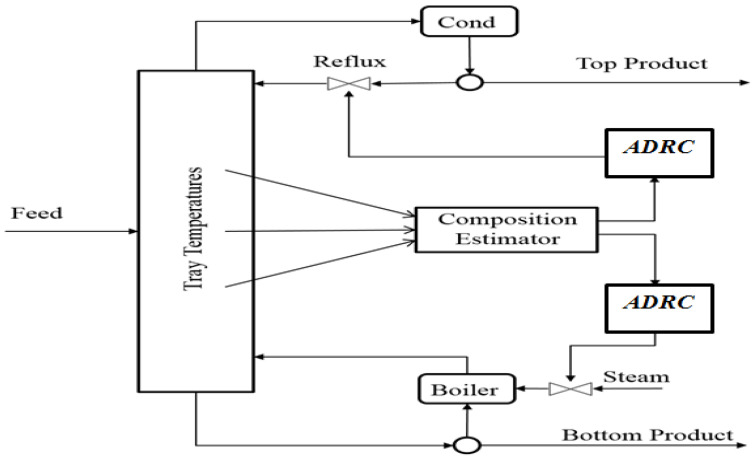
Inferential ADRC control of product compositions.

In the product composition control of this distillation column, the manipulated variables for top and bottom compositions are reflux flow rate (L) and steam flow rate (V) to the reboiler, respectively. The secondary measurements, which are tray temperature measurements, are fed to the PCR soft sensors to estimate the top and bottom product compositions. Then, the estimations are used in feedback control to the ADRC controller, as shown in Figure 9. The performance of both ADRC and inferential control was investigated through simulation. The following disturbances in the form of step changes were applied to the simulated column: the feed composition was increased by 15% at the 600th minutes and the feed flow rate was increased by 15% at the 1200th minutes. Moreover, series setpoints changes were applied to both top and bottom product compositions.

The inferential ADRC control strategy is compared with single tray temperature control and composition analyzer-based control. Through investigating the data presented in Figure 4 and Figure 5, it was found that the temperature of the 8th tray (from the bottom column) has the largest correlation coefficient with the top product composition and the temperature of the 2nd tray has the largest correlation coefficient with the bottom product composition. Hence, temperatures of the 2nd and the 8th trays were controlled to indirectly control the bottom and top product compositions respectively. Temperatures at the 2nd and the 8th trays corresponding to the top composition of 93% and the bottom composition of 7% are 85.9 °C and 70.5 °C, respectively. Hence, the setpoints for the 2nd and the 8th tray temperatures were set at 85.9 °C and 70.5 °C, respectively. Temperature setpoints corresponding to other product compositions were identified from simulated process operation data. In the product composition analyzer-based composition control, a 10 min measurement delay was assumed. For both cases, multi-loop PI controllers were used.

**Figure 10:**
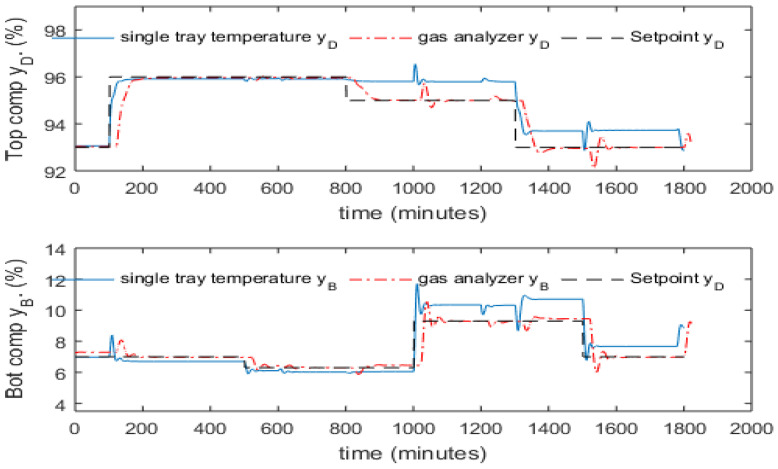
Control performance of tray temperature control and composition analyzer-based control.

Figure 10 shows the control performance of tray temperature control and composition analyzer-based control. The solid, dash-dotted, and dashed lines represent the response of the single tray temperature control, composition analyzer-based control, and the desired set-point signal. It can be seen from this figure that composition analyzer-based control has sluggish response and due to the large measurement delay, the controller has been significantly de-tuned to ensure stability. In the tray temperature control scheme, significant static control offsets exist in both product compositions especially the bottom product composition. This is due to the fact that the relationship between the single tray temperature and product compositions can be significantly affected by the variation of process operating condition such as setpoint changes and the presence of disturbances.

**Figure 11:**
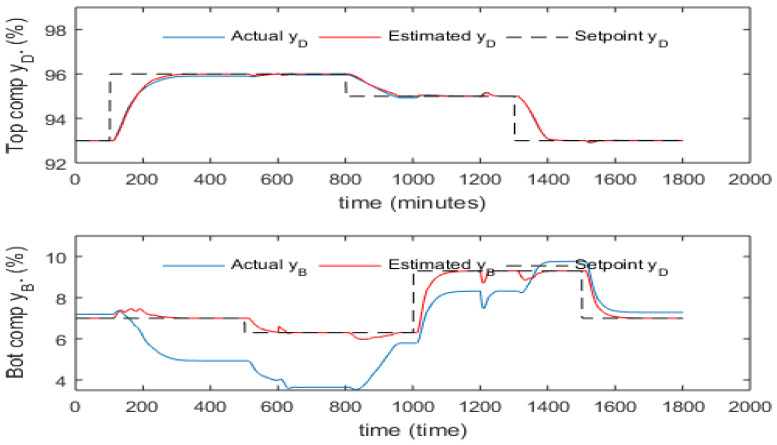
Responses of actual and estimated product compositions under inferential ADRC with static PCR model (without mean updating).

**Figure 12:**
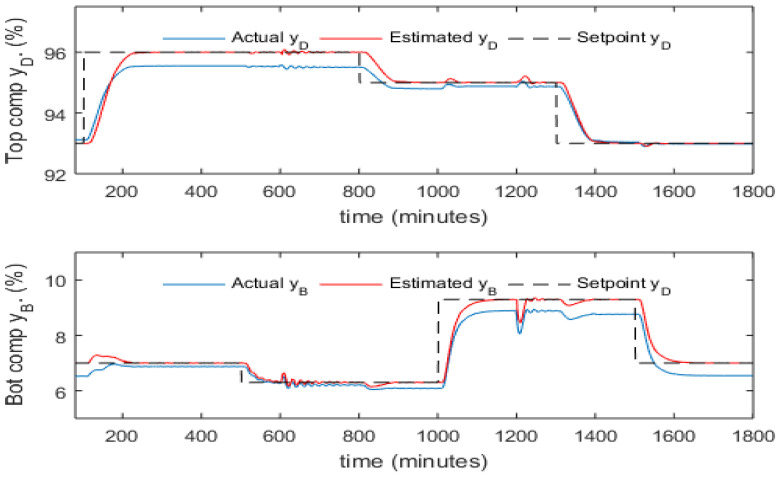
Responses of actual and estimated product compositions under inferential ADRC with 5th order dynamic PCR models (without mean updating).

Figure 11 shows the setpoint tracking and disturbance rejection performance of inferential ADRC with static PCR model across a broad range of setpoint changes, feed flow rate and feed composition disturbances. The setpoint signal was smoothed by TPG to avoid the undesired overshoot. It can be seen that the top composition is controlled quite well with small static control offsets, but large static control errors exist for the bottom product composition. The static control errors are due to the errors of the PCR models which can get worse when operating condition changes such setpoint change and/or disturbance changes. Figure 12 shows the setpoint tracking and disturbance rejection performance of inferential ADRC with the 5th order dynamic PCR model for the same setpoint changes, feed flow rate and feed composition disturbances, as shown in Figure 11. It can be seen the control performance improved under the dynamic PCR model. However, static control offsets still exist.

**Figure 13:**
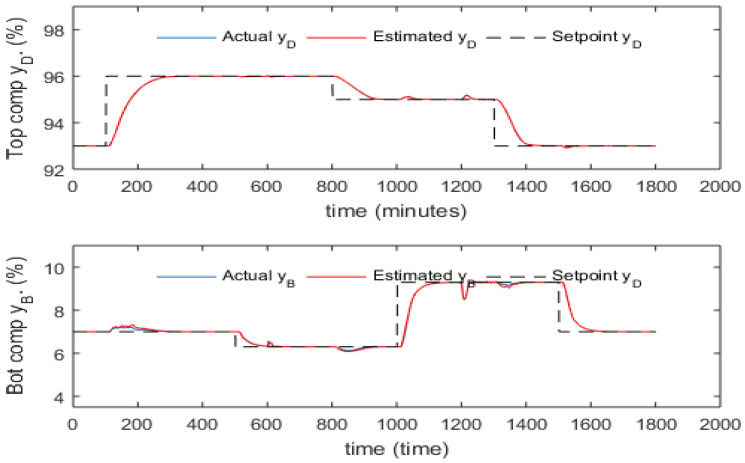
Responses of actual and estimated product compositions of static inferential ADRC (with mean updating).

**Figure 14:**
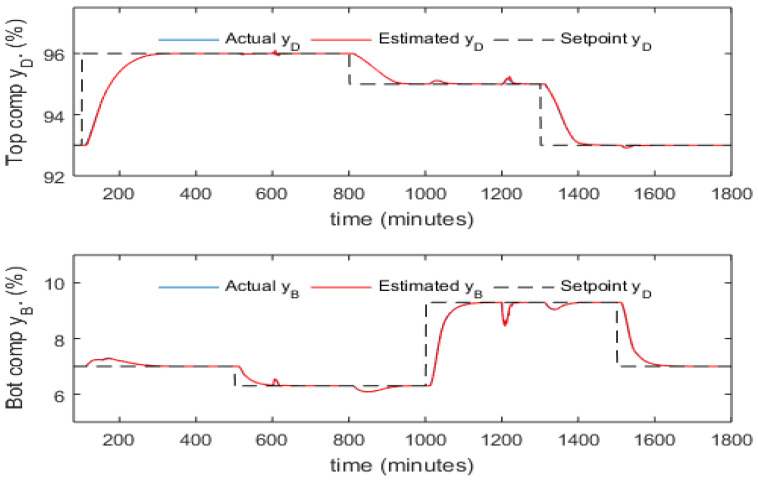
Responses of actual and estimated product compositions under inferential ADRC with 5th order dynamic PCR models (with mean updating).

To overcome the static control offset issue due to the variation in process operating conditions, the intermittent process variable mean updating strategy proposed by Zhang [24] is used here. When a new steady state is reached, the static values of product compositions and tray temperatures are used to replace the current mean values of these variables in the PCR models. It can be seen here that only intermittent product composition measurements are required. Figure 13 and Figure 14 present the control performance with mean updating technique. It can be noticed from these figures that the mean updating technique is an efficient technique for significantly reducing the static control offsets. Moreover, the SSE of control errors has been reduced dramatically after using the mean updating technique, as shown in Table 4.

It can be seen from Figure 13 and Figure 14 that the resulting control off-sets and steady state model estimation bias have been eliminated successfully through the mean updating technique. Moreover, it can be noticed from Table 4 that the dynamic PCR model has much smaller estimation off-sets than the static PCR model when the operating condition changed. This leads to a result that the dynamic PCR model is more robust than the static PCR model to process operating condition variations. As a result, the dynamic inferential ADRC scheme gives better control performance than the static inferential ADRC.

## 7. Conclusions

Inferential ADRC control schemes with static and dynamic PCR models are proposed for product composition control in distillation columns. Inferential estimation models for product compositions are developed from process operational data using PCR. The estimated product compositions are used as the controlled variables in the ADRC controller. Mean updating technique is used to eliminate the steady state model estimation bias and the resulting control off-sets. The proposed control method is applied to a simulated methanol–water separation column. Simulation results indicate the effectiveness and success of the proposed dynamic inferential ADRC control method over the static inferential ADRC control method. As a future work, the inferential ADRC control method will be applied to high purity distillation columns and heat integrated distillation columns.

## Figures and Tables

**Table 1:** Nominal distillation column operation data.

Variables	Nominal Values
Top composition (*y*_1_)	93% (wt) methanol
Bottom composition (*y*_2_)	7% (wt) methanol
Reflux flow rate (*u*_1_)	10.108 g/s
Steam flow rate (*u*_2_)	13.814 g/s
Feed composition (*d*_1_)	50.12% (wt) methanol
Feed flow rate (*d*_2_)	18.23 g/s

**Table 2:** SSE on training and testing data for static PCR models with different numbers of principal components.

No. of PCs	Top Composition		Bottom Composition	
	Training Data	Testing Data	Training Data	Testing Data
1	410.00	230.00	1400	280.30
2	32.00	10.00	679.90	82.27
3	31.00	10.00	89.39	19.71
4	4.00	0.78	68.10	8.50
5	3.50	0.48	49.35	6.71
6	3.15	0.32	40.86	5.45
7	3.14	0.32	35.64	6.43
8	3.07	0.36	27.82	3.36
9	2.93	0.38	20.21	2.66
10	2.85	0.34	17.86	1.94

**Table 3:** Number of principal components and SSE on testing data of different dynamic PCR models.

Model Orders	Model Output	SSE	No. of Principal Components
1	Top composition	0.662	11
	Bot composition	13.04	11
2	Top composition	0.361	14
	Bot composition	9.958	7
3	Top composition	0.045	32
	Bot composition	2.970	7
4	Top composition	0.140	50
	Bot composition	2.542	7
5	Top composition	0.122	17
	Bot composition	1.323	7
6	Top composition	0.145	42
	Bot composition	4.722	8
7	Top composition	0.141	54
	Bot composition	3.958	8

**Table 4:** SSE of different control schemes.

Control Schemes		Top Comp.	Bottom Comp.
Inferential ADRC with static PCR model	Without mean updating	54,542	6946.9
	With mean updating	1.6889	1.8309
Inferential ADRC with 5th order dynamic PCR model	Without mean updating	165.52	219.59
	With mean updating	0.1856	0.1551

## Data Availability

Not applicable.

## Appendix A

Model parameters of the 5th order dynamic PCR models are given in Table A1 (for top composition) and Table A2 (for bottom composition).

**Table A1:** Model parameters for top composition.

	*t*	*t* − 1	*t* − 2	*t* − 3	*t* − 4	*t* − 5
*T*_1_	−0.037	0.006	0.077	0.091	0.039	−0.151
*T*_2_	0.012	−0.039	−0.030	−0.061	0.031	−0.001
*T*_3_	0.115	0.059	0.031	−0.021	−0.002	−0.030
*T*_4_	0.051	0.014	−0.003	−0.035	−0.009	−0.020
*T*_5_	0.046	−0.022	−0.021	−0.044	−0.052	−0.016
*T*_6_	−0.083	−0.045	0.056	0.068	0.065	0.016
*T*_7_	−0.138	−0.069	0.020	0.044	0.071	0.055
*T*_8_	−0.171	−0.110	−0.042	−0.023	0.004	0.007
*T*_9_	−0.175	−0.103	−0.015	0.013	0.068	0.100
*T*_10_	−0.219	−0.146	−0.088	−0.071	−0.047	−0.017

**Table A2:** Model parameters for bottom composition.

	*t*	*t* − 1	*t* − 2	*t* − 3	*t* − 4	*t* − 5
*T*_1_	−0.569	−0.453	−0.307	−0.140	0.032	0.191
*T*_2_	−0.122	−0.084	−0.037	0.042	0.154	0.261
*T*_3_	0.056	0.052	0.047	0.060	0.100	0.142
*T*_4_	0.019	−0.004	−0.041	−0.076	−0.093	−0.097
*T*_5_	0.083	0.059	0.020	−0.033	−0.084	−0.122
*T*_6_	0.113	0.065	0.016	−0.028	−0.005	−0.062
*T*_7_	0.002	−0.027	−0.047	−0.053	−0.041	−0.015
*T*_8_	0.032	0.014	0.004	0.007	0.026	0.055
*T*_9_	−0.008	−0.033	−0.048	−0.048	−0.027	0.008
*T*_10_	0.017	0.001	−0.004	0.003	0.028	0.067

## References

[1] Fazlali A., Hosseini S., Yasini B., Moghadassi A. (2009). Optimization of operating conditions of distillation columns: An energy saving option in refinery industry. Songklanakarin J. Sci. Technol..

[2] Hewitt G., Quarain J., Morell M. (1999). More efficient distillation. Chem. Eng..

[3] Kiss A., Bildea C. (2011). A control perspective on process intensification in dividing-wall columns. Chem. Eng. Process. Process Intensif..

[4] Kiss A. (2013). Distillation technology-still young and full of breakthrough opportunities. J. Chem. Technol. Biotechnol..

[5] Brockett R. (2001). New Issues in the Mathematics of Control.

[6] Han J. (1998). Auto-disturbances-rejection controller and its application. Control Decis..

[7] Han J. (1995). A class of extended state observers for uncertain systems. Control Decis..

[8] Han J. (2008). Active Disturbance Rejection Control Technique—The Technique for Estimating and Compensating the Uncertainties.

[9] Han J. (2009). From PID to active disturbance rejection control. IEEE Trans. Ind. Electron..

[10] Gao Z. Scaling and bandwidth-parameterization based controller tuning. Proceedings of the 2003 American Control Conference.

[11] Gao Z. Active disturbance rejection control—A paradigm shift in feedback control system design. Proceedings of the 2006 American Control Conference.

[12] Gao Z., Huang Y., Han J. An alternative paradigm for control system design. Proceedings of the 40th IEEE Conference on Decision and Control.

[13] Mejdell T., Skogestad S. (1991). Estimation of distillation compositions from multiple temperature measurements using partial-least-squares regression. Ind. Eng. Chem. Res..

[14] Kister H. (1990). Distillation Operation.

[15] Fortuna L., Graziani S., Xibilia M. (2005). Soft sensors for product quality monitoring in debutanizer distillation columns. Control Eng. Pract..

[16] Tham M., Montague G., Morris A.J., Lant P. (1991). Soft-sensors for process estimation and inferential control. J. Process Control.

[17] Weber R., Brosilow C. (1972). The use of secondary measurements to improve control. AIChE J..

[18] Joseph B., Brosilow C. (1978). Inferential control of processes: Part III. Construction of optimal and suboptimal dynamic estimators. AIChE J..

[19] Corrigan J., Zhang J. (2021). Developing accurate data-driven soft-sensors through integrating dynamic kernel slow feature analysis with neural networks. J. Process Control.

[20] Kresta J., Marlin T., MacGregor J. (1994). Development of inferential process models using PLS. Comput. Chem. Eng..

[21] Mejdell T., Skogestad S. (1991). Composition estimator in a pilot-plant distillation column using multiple temperatures. Ind. Eng. Chem. Res..

[22] Mejdell T., Skogestad S. (1993). Output estimation using multiple secondary measurements: High-purity distillation. AIChE J..

[23] Zhang J. Inferential feedback control of distillation composition based on PCR and PLS models. Proceedings of the American Control Conference 2001.

[24] Zhang J. (2006). Offset-free inferential feedback control of distillation compositions based on PCR and PLS models. Chem. Eng. Technol..

[25] Al-Kalbani F., Al Hosni S.M., Zhang J. Active disturbance rejection control of a methanol-water separation distillation column. Proceedings of the 8th IEEE GCC Conference and Exhibition.

[26] Dias T., Oliveira R., Saraiva P.M., Reis M.S. (2022). Linear and non-linear soft sensors for predicting the research octane number (RON) through integrated synchronization, resolution selection and modelling. Sensors.

[27] Li Y., Yang C., Sun Y. (2022). Sintering quality prediction model based on semi-supervised dynamic time feature extraction framework. Sensors.

[28] Severino A.G.V., de Lima J.M.M., de Araújo F.M.U. (2022). Industrial soft sensor optimized by improved PSO: A deep representation-learning approach. Sensors.

[29] Paepae T., Bokoro P.N., Kyamakya K. (2022). A virtual sensing concept for Nitrogen and Phosphorus monitoring using machine learning techniques. Sensors.

[30] Al-Kalbani F., Zhang J. Inferential active disturbance rejection control of a distillation column. Proceedings of the 9th IFAC International Symposium on Advanced Control of Chemical Processes (ADCHEM2015).

